# Environmental sources of *Cryptococcus neoformans* species complex in Kampala, Uganda: A preliminary study

**DOI:** 10.1371/journal.pone.0329947

**Published:** 2025-08-18

**Authors:** Beatrice Achan, Abel Wembabazi, Tonny Luggya, Innocent Robert Ebwongu, Benson Musinguzi, Herbert Itabangi, Henry Kajumbula, David Meya

**Affiliations:** 1 Department of Medical Microbiology, School of Biomedical Sciences, College of Health Sciences, Makerere University, Kampala, Uganda; 2 Department of Dentistry, School of Dentistry, College of Health Sciences, Makerere University, Kampala, Uganda; 3 Department of Immunology and Molecular Biology, School of Biomedical Sciences, College of Health Sciences, Makerere University, Kampala, Uganda; 4 Department of Microbiology and Immunology, Faculty of Health Sciences, Busitema University, Mbale, Uganda; 5 Department of Medicine, School of Medicine, College of Health Sciences, Makerere University, Kampala, Uganda; Gulu University, UGANDA

## Abstract

**Objectives:**

We aimed to determine the environmental sources of *C. neoformans* and *C. gattii* species complexes in Uganda.

**Methods:**

One hundred fifty (n = 150) environmental specimens of chicken droppings, marabou stork bird droppings and eucalyptus tree barks in Uganda were examined phenotypically. The specimens were grown on Sabouraud dextrose agar and the colonies examined by India ink microscopy, urea hydrolysis and formation of blue colonies on chromogenic L-Canavanine glycine blue bromothymol blue agar of *C. gattii species complex*

**Results:**

The prevalence of *C. neoformans* species complex was 4% (6/150). Of the positive environmental samples from which *C. neoformans* species complex were isolated; the predominant source was marabou stork droppings; 8% (2/25) followed by eucalyptus tree barks; 3.96% (4/101). However, there was no *Cryptococcus gattii* species complex; 0% (0/150).

**Conclusion:**

In Uganda, the environmental sources of *C. neoformans* species complex are marabou stork droppings and eucalyptus tree barks, however, there seems to be no *Cryptococcus gattii* species complex.

## Introduction

*Cryptococcus neoformans* species complex is the leading cause of fungal attributable mortality in HIV/AIDS [1]. The basidiospores of *C. neoformans* species complex inhaled from environmental sources such as wood debris and birds’ droppings rarely cause disease in immunocompetent individuals [2]. However, disseminated disease with a predilection for the meninges to cause cryptococcal meningitis (CM) occurs due to abrogation of cell-mediated immunity following depletion of CD4 + T cells (< 100/mL) [3]. CM is usually the first AIDS-defining illness in HIV infection [3]. The annual global burden of CM is estimated at 152,000 incident cases which results in 112, 000 cryptococcal-related of which 70% occur in Sub-Saharan Africa AIDS [1]. CM is so highly fatal that even with ART and antifungal therapy, the 6-month survival remains ≤ 40% in routine care [4,5].

Two closely related opportunistic yeasts; *Cryptococcus neoformans* and *C. gattii* species complexes cause CM [6]. The two *Cryptococcus* species complexes are basidiomycetous encapsulated yeast species which are likely inhaled from the environment However, the two closely related species complexes differ in ecology and geographic distribution [7].

*C. neoformans* species complex which causes CM in immunocompromised individuals, for example, in patients with cancer, solid organ transplant, and HIV/AIDS is the most prevalent and globally distributed [8,9]. *C. neoformans* species complex is ubiquitous in the environment, where it is usually associated with avian droppings [10]. The sibling species *Cryptococcus gattii* species complex*,* which induces cryptococcal meningitis more often than *C. neoformans* species complex in immunocompetent patients, has traditionally shown a geographic restriction to tropical and subtropical regions [11,12] and [13–16]. *C. gattii* species complex is often associated with the red gum trees; *Eucalyptus* species (*Eucalyptus camaldulensis*) [17–19]. More than 50 tree species have yielded *C. gattii* species complex [20], especially, in Egypt and Tunisia [21,22], Cassia tree in Kenya [10] and Almond tree in Tunisia [22]. In Zambia, environmental sampling identified *C. neoformans species complex* from Zambezi Mopane woodlands and *C. gattii* species complex primarily recovered from Central Miombo woodlands [23]. Since these fungi are acquired from nature, environmental sampling is key to understanding their ecological niches. However, the source of *Cryptococcus* in nature has not yet been identified in Uganda. Therefore, we aimed to screen *C. neoformans* and *C. gattii* species complexes environmental isolates from *Eucalyptus* tree barks, Marabou stork bird droppings and domestic chicken droppings in Kampala, Uganda.

## Materials and methods

### Study design and duration

A preliminary study was conducted from January 2022 to December 2022.

### Study site

The study site was the Kampala metropolitan area, Uganda.

### Sample type

Environmental samples which were obtained from chicken droppings, marabou stork bird droppings and *Eucalyptus* tree barks in Kampala Metropolitan area were processed at the Clinical Microbiology, and Mycology laboratories of Makerere University College of Health Sciences, Kampala, Uganda.

### Sample size

One hundred fifty (n = 150) environmental samples were obtained from chicken droppings, Marabou stork bird droppings and *Eucalyptus* tree barks in Kampala metropolitan area, Uganda (S1 Data). A positive control for *C. neoformans* species complex was included. The origin of the positive *C. neoformans* species control used was a patient’s cerebrospinal fluid which was identified through phenotypic-based positive India ink stain, growth of cream mucoid colonies on Sabouraud Dextrose agar and positive urease hydrolysis. A previous study in Uganda showed that 99% of *C. neoformans* isolates are urease positive [24]. However; we did not have a positive control for *C. gattii* species complex.

### Sampling technique

Sampling was performed by swabs of bird, marabou stork droppings and at the under surfaces of tree barks or sites not directly exposed to the sunlight. For each sample; two swabs were collected until the sample size of 150 environmental samples was achieved. The samples were collected from chicken droppings, marabou stork bird droppings and *eucalyptus* tree barks in Kampala metropolitan area, Uganda.

### Laboratory procedures

#### Sample collection and transport.

Environmental samples were collected from chicken droppings, marabou stork bird droppings and *Eucalyptus* tree barks in Kampala metropolitan area, Uganda, using sterile moist cotton swabs. The swabs were transported in amies transport medium to the testing laboratory within 24 hours.

#### Culture and identification.

Each swab was streaked onto Sabouraud Dextrose Agar plate and cultured at 37^o^. After 48 hours, the first grown colonies were analysed phenotypically while the plates without colonies were incubated and examined regularly up to 4 weeks. Firstly, the colonies were stained with India Ink and examined under x40 objective for the presence of the polysaccharide capsule then streaked onto urea agar and incubated for 48 hours to observe the pink colour of urease positive isolates of *C. neoformans* and *C. gattii* species complexes. Lastly, urease positive pure colonies were streaked on CGB agar which is a differential medium with bromothymol blue indicator which is used to identify *C. gattii* that form blue colonies. CGB agar plate preparation was done as described here [19].

#### Ethical consideration.

Ethical approval was sought from the Makerere University College of Health Sciences’ School of Biomedical Sciences’ Higher Degrees’ Research and Ethics Committee (SBS 639) and the Uganda National Council for Science and Technology (HS1127ES) to carry out the study in Uganda.

#### Quality assurance.

Standard procedures were followed while preparing the reagents and media (agar) and performing the experiments. Their accuracy was double-checked by performing quality control. The research assistants underwent a basic laboratory training before the start of the study. All activities were under the supervision of the Principal Investigator.

## Results

**Fig 1:**
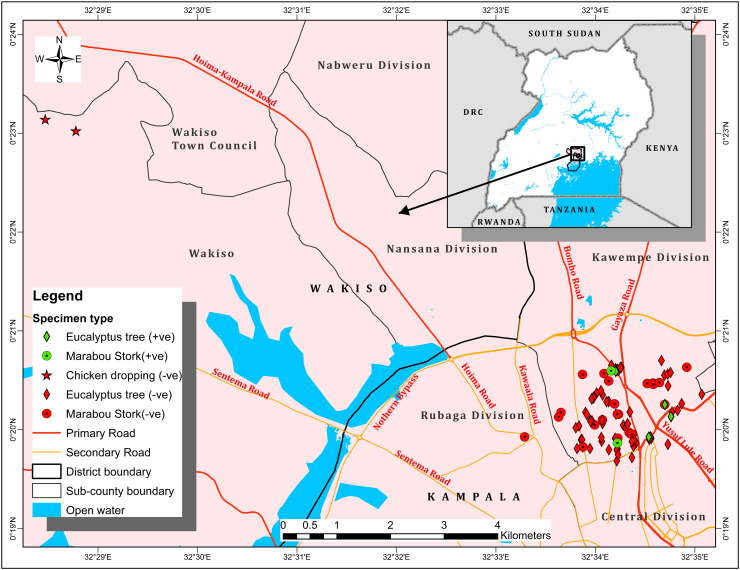
Kampala Metropolitan area showing where all the 150 samples were collected from. The GPS locations of Kampala metropolitan areas where samples of eucalyptus tree bark, marabou stork and chicken droppings were collected are shown in the map of Uganda. The inset of Kampala metropolitan areas within the map of Uganda is marked with a square and an arrow used to show the display.

In this study, 150 environmental samples from different sites within Kampala Metropolitan area were analysed for *Cryptococcus* strains. A positive control for *C. neoformans* species complex was included; however, we did not have a positive control for *C. gattii* species complex. The samples included swabs from 24 chicken droppings, 25 Marabou stork droppings and 101 eucalyptus tree bark swabs (Table 1). Of the N = 150 specimens, 4% (6/150) isolates (0 Chicken, 2 Marabou stork and 4 eucalyptus tree barks) were India ink and urease test positive and thus, confirmed as *C. neoformans* species complex (Tables 2 and 3, Fig 1). However, all the isolates were negative on CGB agar which suggests that no *C. gattii* species complex was present (Table 3).

**Table 1:** Growth of environmental specimens on *SAB agar (n = 150).

Type of environmental sites/specimens	Number of specimens, n (%)	Growth on SAB agar, n (%)
Eucalyptus tree barks	101 (67.3)	101 (67.3)
Marabou stork droppings	25 (16.7)	25 (16.7)
Chicken droppings	24 (16)	24 (16)
Total	**150 (100)**	**150 (100)**

Environmental specimens of eucalyptus tree barks, marabou stork and, chicken droppings were streaked on SAB agar, incubated at 35-37°C with ambient air for 48 h.

*SAB: Sabouraud Dextrose Agar.

**Table 2:** India ink stain of positive culture on SAB agar (n = 150).

Environmental sites/specimens	Negative India ink stain, n (%)	Positive India ink stain, n (%)
Eucalyptus tree barks	97 (64.67)	4 (2.67)
Marabou stork droppings	23 (15.33)	2 (1.33)
Chicken droppings	24 (16.00)	0 (0.00)
Total	**144 (96.00)**	**6 (4.00)**

India ink stain of positive culture of environmental specimens on SAB agar (n = 6).

**Table 3:** Urease test, blue colonies on CGB agar and species names of India ink positive isolates (n = 6).

Environmental sites/specimens	Positive Urease test, n (%)	Blue colonies on CGB agar, n (%)	Species names
Eucalyptus tree barks	4 (2.67)	0 (0.00)	*Cryptococcus neoformans*
Marabou stork droppings	2 (1.33)	0 (0.00)	*Cryptococcus neoformans*
Total	**6 (4.00)**	**0 (0.00)**	

Urease test and formation of blue colonies on the CGB agar of India ink positive colonies (n = 6).

## Discussion

This study reports the environmental screening report for *Cryptococcus* species in Uganda where 70% of all meningitis cases especially in immunocompromised patients are due to cryptococcal meningitis [2]. We found 4% (150) environmental prevalence of *C. neoformans* species complex, however, no *C. gattii* species complex was isolated. This suggests that there was no *C. gattii* species complex in the sampled specimens in Uganda’s environment. Our finding is in consistence with our previous publication in which we demonstrated *C. neoformans* species complex infections but no *C. gattii* species complex infections among Ugandan HIV-infected patients presenting with cryptococcal meningitis [24]. *C. neoformans* species complex were predominantly identified in marabou stork droppings at 8% (2/25) followed by eucalyptus tree barks; 3.96% (4/101) similar to other environmental studies reported in the nearby Kenya [10]. However, no *C. neoformans* species complex was isolated from chicken droppings.

*C. gattii* species complex is traditionally associated with the red gum trees, *Eucalyptus* species [19,22], and has shown a geographic restriction to tropical and subtropical regions, however, in this study, none was identified from the samples analyzed. Several other studies have reported environmental presence of *C. neoformans/C. gattii* species complexes globally [11,19,22].

In this study, samples were analyzed phenotypically as described above in the culture and identification section because of the limited resources within the scope of this study. However, recent studies have employed genetic and molecular means in determining genotypic diversity of *C. neoformans*/*C. gattii* species complexes [5,18,25] which would give a better accuracy of identification.

This study highlights the isolation of *C. neoformans* species complex from eucalyptus tree barks and marabou stork droppings. Eucalyptus tree species grow across the whole country in both cooler and hotter areas and are choice for many commercial farmers because it is multipurpose, fast-growing and has a ready market. This followed decimation of natural forests and woodlands across Africa which could no longer keep pace with the required demand for forest cover, energy usage, timber and construction work associated urbanization [26]. Marabou storks are common in urban areas especially around the garbage collection sites and markets due to their vulturous nature of diet. The finding of the urban ecological niches of *C. neoformans* species complex in this study is in agreement with the previous study which showed that up to 93.5% (n = 200) of the Infectious Diseases Institute’s recruited patients with AIDS-associated cryptococcal meningitis were from Uganda’s central region the capital city; Kampala, is located [24]. Furthermore, the previous clinical study showed that 45% of the patients with cryptococcal meningitis were in the age category 30–39 years old [24], which represents the most physically productive age group that would be involved with urban economic activities such as building using timber from eucalyptus tree. This calls for safety measures when dealing with Marabou stork and farmers/ individuals who work in eucalyptus tree plantations.

However, there could be many more undocumented environmental reservoirs of *C. neoformans* species complex*/C. gattii* species close to or within human habitation which might be a significant exposure risk factor for the high prevalence of cryptococcal meningitis especially among the immunocompromised patients in Uganda.

### Study limitations and strengths

Limitations of the study include the small sample size, limited geographical coverage of sample collection sites and use of phenotypic assays. A significant strength of the study is that this is the first report of environmental presence of *Cryptococcus neoformans* species complex in Uganda.

## Conclusion

The prevalence of *C. neoformans* species complex in Uganda’s environment is 4%, predominantly, in marabou stork droppings. However, there seems to be no species belonging to the *C. gattii* species complex in our pool of samples. We recommend further genetic and molecular studies on a larger number of isolates from increased numbers of different environmental specimens which may unearth the distribution of the of a *C. neoformans* and *C. gattii* species complexes in Uganda.

## Supporting information

S1 Data Environmental specimen types that were collected.
(XLSX)
